# Maternal Disability and Emergency Department Use for Infants

**DOI:** 10.1001/jamanetworkopen.2025.8549

**Published:** 2025-05-05

**Authors:** Hilary K. Brown, Yona Lunsky, Kinwah Fung, Maria Santiago-Jimenez, Andi Camden, Eyal Cohen, Joel G. Ray, Natasha R. Saunders, Deanna Telner, Catherine E. Varner, Simone N. Vigod, Jennifer Zwicker, Astrid Guttmann

**Affiliations:** 1 Department of Health & Society, University of Toronto Scarborough, Toronto, Ontario, Canada; 2 Dalla Lana School of Public Health, University of Toronto, Toronto, Ontario, Canada; 3 Women’s College Research Institute, Women’s College Hospital, Toronto, Ontario, Canada; 4 ICES, Toronto, Ontario, Canada; 5 Azrieli Adult Neurodevelopmental Centre, Centre for Addiction & Mental Health, Toronto, Ontario, Canada; 6 Department of Psychiatry, University of Toronto, Toronto, Ontario, Canada; 7 Edwin SH Leong Centre for Healthy Children, University of Toronto, Toronto, Ontario, Canada; 8 Hospital for Sick Children, Toronto, Ontario, Canada; 9 Department of Pediatrics, University of Toronto, Toronto, Ontario, Canada; 10 Li Ka Shing Knowledge Institute, St Michael’s Hospital, Toronto, Ontario, Canada; 11 Department of Family and Community Medicine, University of Toronto, Toronto, Ontario, Canada; 12 Schwartz/Reisman Emergency Medicine Institute, Toronto, Ontario, Canada; 13 Department of Emergency Medicine, Mount Sinai Hospital, Toronto, Ontario, Canada; 14 Faculty of Kinesiology, University of Calgary, Calgary, Alberta, Canada

## Abstract

**Importance:**

Infants have among the highest rates of emergency department (ED) visits of all age groups. Women with disabilities experience significant social disparities, may experience barriers accessing child primary care, and may seek care in the ED.

**Objective:**

To compare ED use among newborns and infants (hereafter infants) of women with or without disabilities, overall and by timing and acuity of the ED visit.

**Design, Setting, and Participants:**

This population-based cohort study analyzed data for all live-born infants in Ontario, Canada, born to women with or without a disability from April 1, 2008, to March 31, 2021. Data were analyzed March 2023 to October 2024.

**Exposure:**

Maternal physical, sensory, or intellectual or developmental disability or multiple disabilities status was ascertained using diagnostic algorithms applied to health care encounters before delivery.

**Main Outcomes and Measures:**

The main outcome was any ED visit between an infant’s hospitalization discharge date and their first birthday, and by timing and acuity. Cox proportional hazards regression was used to generate hazard ratios (HRs) for the association between maternal disability and the main outcome, adjusted for sociodemographic characteristics and infant sex and year of birth.

**Results:**

Of 1 596 932 total infants, there were 139 698 (8.7%) born to women with a physical disability, 48 112 (3.0%) to women with a sensory disability, 2547 (0.2%) to women with an intellectual or developmental disability, and 10 312 (0.6%) to women with multiple disabilities. The remaining 1 396 263 infants (87.4%) were born to a woman without a recognized disability. Among women without a disability, 558 965 infants (40.0%) had an ED visit in the first year of life (incidence rate, 1.11 per 1000 person-days). Compared with this referent group, infants born to a woman with a disability of a physical (46.9%; 1.30 visits per 1000 person-days; adjusted HR [AHR], 1.14 [95% CI, 1.13-1.15]), sensory (45.2%; 1.25 visits per 1000 person-days; AHR, 1.09 [95% CI, 1.07-1.10]), or intellectual or developmental (55.4%; 1.55 visits per 1000 person-days; AHR, 1.24 [95% CI, 1.17-1.30]) nature or with multiple disabilities (51.0%; 1.42 visits per 1000 person-days; AHR, 1.18 [95% CI, 1.15-1.22]) were more likely to have an ED visit. Similar patterns were observed for ED visits at fewer than 28 days from delivery, from 28 to 365 days, and for ED visits for high-acuity, moderate-acuity, and low-acuity reasons.

**Conclusions and Relevance:**

In this population-based cohort study, infants of women with disabilities were more likely to use the ED, indicating a need for accessible family supports and improved early child primary care access.

## Key Points

**Question:**

Do women with a disability more often seek emergency department (ED) care for their infants compared with women without a disability?

**Findings:**

In this cohort study of 1 596 932 infants born in Ontario, Canada, infants of women with physical (46.9%), sensory (45.2%), or intellectual or developmental (55.4%) disability or multiple disabilities (51.0%) had higher ED use than those without a disability (40.0%).

**Meaning:**

Among women with a disability, preventive efforts may be needed to reduce their infant ED use, including tailored family supports to address social disparities and improve access to primary care.

## Teaser

This cohort study compares emergency department use rates overall and by timing and acuity for infants born to women with or without a disability.

## Introduction

Infants younger than 1 year have among the highest rates of emergency department (ED) visits of all age groups and account for one-fifth of pediatric ED visits.^1,2^ Respiratory illness and fever are the most common reason for an infant ED visit, and in newborns younger than 28 days, jaundice, feeding issues, and infection are important contributors.^1,3^ ED visits are also considered indicators of whether an infant has timely access to high-quality primary care, as ED visits could potentially be avoided by adequate preventive hospital care at birth and access to a primary care physician.^3^ In fact, low-acuity ED visits represent 20% of ED visits in the first 3 months of life.^4^ Pediatric ED visits in North America have increased by 30% over the last 15 years^1,5^ and may contribute to escalating health care costs and ED crowding, the latter of which is associated with poor patient satisfaction, adverse outcomes, and staff stress.^6^ ED visits are also disruptive for families.^7^ Identifying families at risk of infant ED visits is thus critical for informing tailored preventive measures, improved continuity of primary care, and appropriate ED supports.

Research has shown that infant ED visits are socially patterned, with higher rates among families experiencing poverty, racism, and other barriers to health care.^3,4,7,8^ Other groups, including infants of women with disabilities, may also have elevated risk. One in 8 births are to women with a physical, sensory, or intellectual or developmental disability.^9^ Women with disabilities experience significant social disparities^10,11,12^ and are themselves at elevated risk of ED visits, overall and for ambulatory care–sensitive conditions^13,14^ for which appropriate primary care could have avoided the need for acute care.^15^ Yet no studies, to our knowledge, have examined ED visits among infants of these women, with the scant literature on health care use in this group, mostly from small clinical studies and surveys, suggesting small differences in well-infant care access^16,17^ and elevated risk of hospitalization among infants of women with an intellectual or developmental disability.^17,18,19,20^ There is a need for population-based data on ED visits among infants of women with disabilities to inform preventive measures, as well as accessible family supports in ED settings and after discharge.

We compared ED visits among infants of women with a physical, sensory, or intellectual or developmental disability with those without a disability, overall and by timing and acuity of the ED visit. Among infants with an ED visit, we also examined the proportion of infants with a follow-up visit with their primary care physician and with repeat ED visits, both within 7 days of the initial ED visit.

## Methods

### Study Design and Data Sources

We conducted a population-based cohort study in Ontario, Canada, following the Strengthening the Reporting of Observational Studies in Epidemiology (STROBE) reporting guideline.^21^ Ontario is Canada’s most populous province (14.7 million residents) and has a universal health care system. We accessed and analyzed data at ICES (formerly the Institute for Clinical Evaluative Sciences), a not-for-profit research institute that houses hospital, outpatient, and sociodemographic data (eTable 1 in Supplement 1),^22^ linked using a unique encoded identifier. ICES is a prescribed entity under Ontario’s Personal Health Information Protection Act (PHIPA). Section 45 of PHIPA authorizes ICES to collect personal health information, without patient consent, for health system planning and evaluation. Projects using data collected by ICES under section 45, and no other data, are exempt from ethics board review. Data use was authorized under section 45 and approved by the ICES Privacy and Legal Office.

### Study Population

We included all live births between April 1, 2008, and March 31, 2021, identified in the MOMBABY dataset, which holds records for hospital births (>98% of births in Ontario).^23^ We excluded newborns discharged to social services after the birth hospital stay because they had unknown periods of separation from their birth parents. We then identified newborns and infants (hereafter infants) born to women with a physical (congenital anomaly, musculoskeletal disorder, neurological disorder, or permanent injury), sensory (hearing or vision impairment), or intellectual or developmental (autism or chromosomal anomalies resulting in intellectual disability) disability or 2 or more of these disabilities (multiple disabilities) using algorithms developed by disability epidemiologists and clinicians to identify disability in administrative data.^24,25^ Disabilities were identified from diagnoses recorded in 2 or more physician visits or at least 1 ED visit or hospitalization between database inception and the infant’s delivery.^24,25,26^ Infants of women without a disability were the reference group.

### Outcome Measures

Our primary outcome was any ED visit in the first year of life. ED visits were identified in the National Ambulatory Care Reporting System dataset and defined as an unscheduled visit by a patient seeking immediate care in a facility staffed by physicians 7 days per week, 24 hours per day. In additional analyses, we examined ED visits by timing (<28 days from delivery, 28-365 days from delivery) and Canadian Triage and Acuity Scale (CTAS) score, which reflects how urgently a patient needs care and the most suitable monitoring level or treatment area (low, CTAS 4-5; moderate, CTAS 3; and high, CTAS 1-2).^27^ We also described the characteristics of the ED visit, including when the visit occurred (during business hours, after hours, or on weekends), primary discharge diagnosis (by *International Statistical Classification of Diseases and Related Health Problems, Tenth Revision* code), and discharge disposition (admission, death, discharge home, left without being seen, or left against medical advice). Finally, for infants with an ED visit, we examined follow-up with the regular primary care clinician^28^ and repeat ED visits, both measured within 7 days of discharge from the index ED visit.^29^

### Covariates

Covariates were infant sex, year of birth, and maternal sociodemographic factors that could impact health care access^3,4,7,8^ and reflect known differences between women with and without disabilities^10,11,12^: age, parity, neighborhood income quintile, rurality, and immigration status. Neighborhood income quintile was measured by linking residential postal code at delivery with census dissemination area–level income data. Rurality was defined as residence in a community with a population of fewer than 10 000 residents. Immigration status, including category (family or economic class immigrant, refugee) and timing (<10 years, ≥10 years), was identified in the Immigration, Refugees and Citizenship Canada Permanent Residents database.

We also described characteristics that might explain disparities in ED visits between infants born to women with or without a disability. Maternal health characteristics were mental illness, substance use disorder (both measured in ≥2 physician visits or ≥1 ED visit or hospital admission less than 2 years before delivery), and stable and unstable chronic conditions (using the Johns Hopkins Adjusted Clinical Groups system, version 10.0).^30^ Infant health characteristics were preterm birth (<37 weeks’ gestation), small for gestational age (<10th percentile), newborn hospital stay more than 72 hours, and complex chronic conditions.^31^ Finally, health care access was characterized according to prenatal care adequacy^32^ and the presence of a regular primary care clinician for the infant (ie, community health center, family physician in a rostered primary care model, pediatrician, family physician not in a rostered model [walk-in clinics or noncomprehensive care], or none).^33^

### Statistical Analysis

We used descriptive statistics to present the baseline characteristics of the cohort, with infants of women with a physical, sensory, or intellectual or developmental disability or multiple disabilities compared with those without disabilities using standardized differences. Standardized differences higher than 0.10 were considered clinically meaningful.^34^

To address the primary objective, we used Cox proportional hazards regression to compare the hazard of any ED visit in the first year of life among infants of women with physical, sensory, intellectual or developmental, and multiple disabilities with that among infants of women without disabilities. We also examined ED visits by timing and acuity. The underlying timescale was anchored on a newborn’s hospitalization discharge date, and infants were censored at their first birthday, death, or migration out of Ontario. Robust standard errors were used to adjust for the statistical dependence of siblings clustered within mothers.^35^ Models were adjusted for maternal age, parity, income quintile, rurality, immigration status, infant sex, and infant year of birth. Maternal health characteristics were added to the models in a second step to test their association with the results.

We used descriptive statistics to report when during the week the ED visit occurred, discharge diagnosis, and discharge disposition. We also undertook 4 additional analyses related to the primary objective. First, we examined the rate of ED visits using an Andersen-Gill model, an extension of the Cox proportional hazards regression model for recurrent events.^36^ Second, we examined the outcome according to the specific subtype of physical, sensory, intellectual or developmental disability or multiple disabilities. Third, we restricted the model to full-term infants of appropriate size for gestational age and without an extended newborn hospital stay or complex chronic condition (a low-risk group). Finally, we restricted the analyses to infants born in 2008 to 2018 (before the COVID-19 pandemic).

To address the secondary objective, we restricted the cohort to infants with at least 1 ED visit. Based on the first ED visit, we then used modified Poisson regression,^37,38^ which allows for the direct estimation of relative risks (RRs), to examine follow-up with a regular primary care physician and repeat ED visits, both within 7 days of discharge.

Analyses using SAS, version 9.4 (SAS Institute Inc) were conducted from March 2023 to October 2024. Statistical significance was defined as a 95% CI excluding 1.

## Results

### Cohort Characteristics

Of 1 596 932 infants included in the analyses, there were 139 698 infants (8.7%) born to women with a physical disability, 48 112 (3.0%) infants born to women with a sensory disability, 2547 infants (0.2%) born to women with an intellectual or developmental disability, 10 312 infants (0.6%) born to women with multiple disabilities, and 1 396 263 infants (87.4%) born to women without a disability. Most women were in the age category of 25 to 34 years. Compared with women without disabilities (eg, 12.8% aged 15-24 years), more women with a sensory (16.3%) or intellectual or developmental (39.0%) disability or multiple disabilities (18.1%) were younger (Table 1; eTable 2 in Supplement 1). Women with intellectual or developmental disabilities (36.4% in lowest income quintile) were more likely than women without disabilities (21.2% in lowest income quintile) to live in low-income neighborhoods. Women with physical (13.3%) and multiple (13.0%) disabilities were more likely than women without disabilities (9.9%) to live in rural areas. All disability groups were less likely to be refugees or immigrants than long-term residents (eg, for intellectual or developmental disabilities, 92.3% long-term residents vs. 71.5% in those without disabilities). Infants of women with intellectual or developmental and multiple disabilities were more likely than infants of women without disabilities to be born preterm (10.5% and 11.8%, respectively, compared with 7.5%) and have a birth hospital stay longer than 72 hours (24.7% and 23.5%, respectively, compared with 15.7%). Infants born to women with an intellectual or developmental disability were more likely than infants born to women without disabilities to be small for gestational age (16.7% compared with 13.2%) and have a complex chronic condition (7.5% compared with 3.9%). Compared with infants of women without disabilities, infants of women with an intellectual or developmental disability were less likely to have adequate prenatal care (32.8% compared with 39.5%), and those of women with multiple disabilities were more likely to have intensive prenatal care (41.8% compared with 34.0%).

**Table 1.** Baseline Characteristics of Infants Born to Women With a Physical, Sensory, or Intellectual or Developmental Disability or Multiple Disabilities and to Women Without a Disability

Variable	Infants, No. (%)				
	Single disability			Multiple disabilities (n = 10 312)	No disability (n = 1 396 263)
	Physical (n = 139 698)	Sensory (n = 48 112)	Intellectual or developmental (n = 2547)		
Sociodemographic characteristic					
Maternal age, y					
15-24	18 897 (13.5)	7859 (16.3)^a^	993 (39.0)^a^	1868 (18.1)^a^	178 782 (12.8)
25-34	84 834 (60.7)	28 733 (59.7)	1185 (46.5)^a^	5918 (57.4)^a^	886 373 (63.5)
35-49	35 967 (25.7)	11 520 (23.9)	369 (14.5)^a^	2526 (24.5)	331 108 (23.7)
Multiparous	80 371 (57.5)	26 303 (54.7)	1392 (54.7)	5782 (56.1)	791 986 (56.7)
Neighborhood income quintile					
1 (lowest)	28 536 (20.4)	9951 (20.7)	926 (36.4)^a^	2494 (24.2)	295 653 (21.2)
2	27 443 (19.6)	9765 (20.3)	577 (22.7)	2080 (20.2)	276 361 (19.8)
3	28 511 (20.4)	9893 (20.6)	436 (17.1)	2167 (21.0)	290 695 (20.8)
4	30 358 (21.7)	10 308 (21.4)	320 (12.6)^a^	1973 (19.1)	295 333 (21.2)
5 (highest)	24 318 (17.4)	8052 (16.7)	274 (10.8)^a^	1567 (15.2)	233 558 (16.7)
Missing	532 (0.4)	143 (0.3)	14 (0.5)	31 (0.3)	4663 (0.3)
Rural region of residence	18 569 (13.3)^a^	5451 (11.3)	314 (12.3)	1341 (13.0)^a^	137 705 (9.9)
Maternal immigrant status^b^					
Recent refugee	1022 (0.7)^a^	377 (0.8)^a^	20 (0.8)^a^	42 (0.4)^a^	29 092 (2.1)
Nonrecent refugee	2448 (1.8)	1039 (2.2)	12 (0.5)^a^	157 (1.5)	28 988 (2.1)
Recent immigrant	4887 (3.5)^a^	2277 (4.7)^a^	61 (2.4)^a^	165 (1.6)^a^	215 684 (15.4)
Nonrecent immigrant	8974 (6.4)	4043 (8.4)	103 (4.0)^a^	574 (5.6)^a^	123 523 (8.8)
Long-term resident	122 367 (87.6)^a^	40 376 (83.9)^a^	2351 (92.3)^a^	9374 (90.9)^a^	998 976 (71.5)
Infant sex					
Male	71 697 (51.3)	24 767 (51.5)	1300 (51.0)	5290 (51.3)	716 089 (51.3)
Female	68 001 (48.7)	23 345 (48.5)	1247 (49.0)	5022 (48.7)	680 174 (48.7)
Maternal health characteristics					
Mental illness <2 y before delivery	26 271 (18.8)^a^	8095 (16.8)^a^	964 (37.8)^a^	2645 (25.6)^a^	168 824 (12.1)
Substance use disorder <2 y before delivery	3281 (2.3)^a^	588 (1.2)	182 (7.1)^a^	377 (3.7)^a^	14 097 (1.0)
Stable chronic condition <2 y before delivery	44 310 (31.7)	14 979 (31.1)	774 (30.4)	3892 (37.7)^a^	394 097 (28.2)
Unstable chronic condition <2 y before delivery	24 296 (17.4)^a^	7643 (15.9)^a^	452 (17.7)^a^	2429 (23.6)^a^	174 557 (12.5)
Infant health characteristics					
Preterm birth^c^	13 101 (9.4)	4309 (9.0)	267 (10.5)^a^	1212 (11.8)^a^	105 269 (7.5)
Small for gestational age^d^	17 186 (12.3)	6318 (13.1)	426 (16.7)^a^	1412 (13.7)	184 316 (13.2)
Birth hospital stay >72 h	27 009 (19.3)	8456 (17.6)	629 (24.7)^a^	2419 (23.5)^a^	219 099 (15.7)
Complex chronic condition	6840 (4.9)	2280 (4.7)	190 (7.5)^a^	716 (6.9)^a^	55 045 (3.9)
Health care characteristics					
Maternal prenatal care adequacy^e^					
None or unknown	3558 (2.5)	1191 (2.5)	66 (2.6)	239 (2.3)	34 674 (2.5)
Inadequate or intermediate	32 649 (23.4)	11 365 (23.6)	712 (28.0)	2261 (21.9)	336 305 (24.1)
Adequate	52 129 (37.3)	18 105 (37.6)	835 (32.8)^a^	3501 (34.0)^a^	550 896 (39.5)
Intensive	51 362 (36.8)	17 451 (36.3)	934 (36.7)	4311 (41.8)^a^	474 388 (34.0)
Infant regular primary care physician					
No regular primary care physician	2184 (1.6)	628 (1.3)	53 (2.1)	170 (1.6)	20 967 (1.5)
General practitioner, no model	11 331 (8.1)	4412 (9.2)	259 (10.2)	934 (9.1)	127 571 (9.1)
Pediatrician	23 711 (17.0)	9253 (19.2)	444 (17.4)	1821 (17.7)	275 308 (19.7)
General practitioner, rostered	100 542 (72.0)	33 085 (68.8)	1713 (67.3)	7180 (69.6)	953 956 (68.3)
Community health center	1930 (1.4)	734 (1.5)	78 (3.1)	207 (2.0)	18 461 (1.3)

^a^ Standardized difference higher than 0.10 comparing women with the stated disability and women without any disability.

^b^ Recent was defined as immigration within 10 years before the index date; nonrecent, as immigration 10 years or more before the index date.

^c^ Preterm birth was defined as less than 37 weeks’ gestation.

^d^ Small for gestational age was defined as less than 10th percentile.

^e^ Prenatal care adequacy categories are defined based on the number and timing of prenatal visits, using R-GINDEX (Kotelchuk).

### Main Analyses

**Figure 1.**
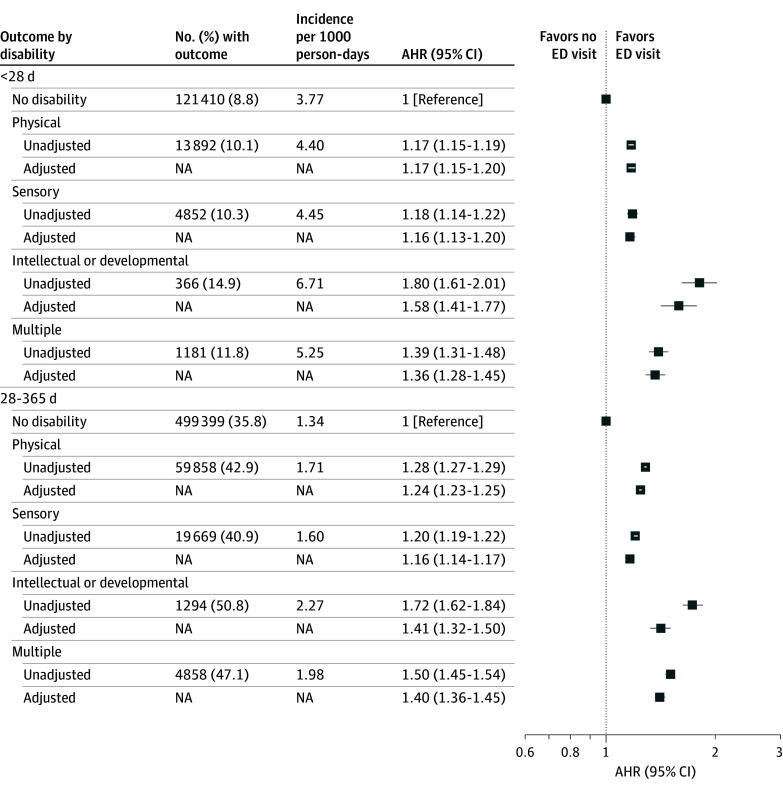
Hazard of an Emergency Department (ED) Visit in the First Year of Life, Comparing Infants of Women With or Without Disabilities, by Timing Model adjusts for infant sex and year of birth and maternal age, parity, neighborhood income quintile, rurality, and immigrant status. Infants who stayed in the hospital for 27 days or more are excluded from the <28-day model. Infants who died at 0 to 27 days are excluded from the 28- to 365-day model. AHR indicates adjusted hazard ratio; NA, not applicable.

**Figure 2.**
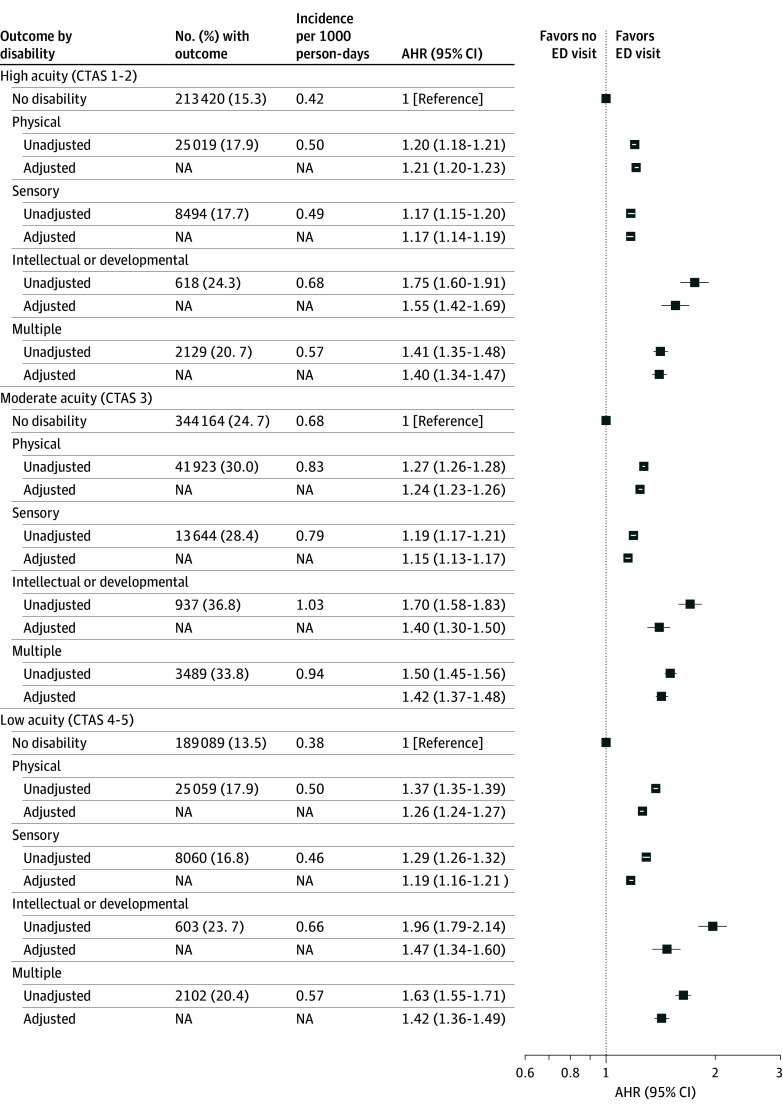
Hazard of Any Emergency Department (ED) Visit in the First Year of Life, Comparing Infants of Women With or Without Disabilities by Canadian Triage and Acuity Scale (CTAS) Score Model adjusts for infant sex and year of birth and maternal age, parity, neighborhood income quintile, rurality, and immigrant status. AHR indicates adjusted hazard ratio; NA, not applicable.

ED visits were common in the first year of life, with 558 965 infants (40.0%) born to women without a disability having such a visit (incidence rate, 1.11 per 1000 person-days of follow-up) (Table 2). Compared with these infants, infants of women with physical (46.9%; incidence rate, 1.30 per 1000 person-days; adjusted hazard ratio [AHR], 1.14 [95% CI, 1.13-1.15]), sensory (45.2%; incidence rate, 1.25 per 1000 person-days; AHR, 1.09 [95% CI, 1.07-1.10]), or intellectual or developmental (55.4%; incidence rate, 1.55 per 1000 person-days; AHR, 1.24 [95% CI, 1.17-1.30]) disability and multiple disabilities (51.0%; incidence rate, 1.42 per 1000 person-days; AHR, 1.18 [95% CI, 1.15-1.22]) were more likely to have an ED visit after adjusting for maternal sociodemographic characteristics, infant sex, and year of birth. Similar results were observed after further adjustment for maternal health characteristics. Similar patterns were observed for ED visits at fewer than 28 days from delivery (eg, 14.9%; incidence rate, 6.71 per 1000 person-days; AHR, 1.58 [95% CI, 1.41-1.77] for intellectual or developmental disability) and 28 to 365 days after delivery (eg, 50.8%; incidence rate, 2.27 per 1000 person-days; AHR, 1.41 [95% CI, 1.32-1.50] for intellectual or developmental disability) (Figure 1) and for high-acuity (eg, 24.3%; incidence rate, 0.68 per 1000 person-days; AHR, 1.55 [95% CI, 1.42-1.69] for intellectual or developmental disability), moderate-acuity (eg, 36.8%; incidence rate, 1.03 per 1000 person-days; AHR, 1.40 [95% CI, 1.30-1.50] for intellectual or developmental disability), and low-acuity (eg, 23.7%; incidence rate, 0.66 per 1000 person-days; AHR, 1.47 [95% CI, 1.34-1.60] for intellectual or developmental disability) visits (Figure 2). There were particularly large disparities for low-acuity visits in unadjusted analyses (eg, AHR, 1.96 [95% CI, 1.79-2.14] for intellectual or developmental disability). Infants of women with or without a disability did not differ greatly with respect to the characteristics of the ED visits, including timing during the week, discharge diagnosis, and discharge disposition (eTable 3 in Supplement 1).

**Table 2.** Hazard of Any Emergency Department Visit in the First Year of Life, Comparing Infants Born to Women With or Without Disabilities

Disability type	Infants, total No.	Infants, No. (%) with outcome	Incidence rate per 1000 person-days	Unadjusted HR (95% CI)	Adjusted HR (95% CI)	
					Model 1^a^	Model 2^b^
None	1 396 263	558 965 (40.0)	1.11	1 [Reference]	1 [Reference]	1 [Reference]
Physical only	139 698	65 496 (46.9)	1.30	1.17 (1.16-1.17)	1.14 (1.13-1.15)	1.12 (1.11-1.13)
Sensory only	48 112	21 744 (45.2)	1.25	1.11 (1.10-1.12)	1.09 (1.07-1.10)	1.07 (1.06-1.09)
Intellectual or developmental only	2547	1410 (55.4)	1.55	1.37 (1.32-1.43)	1.24 (1.17-1.30)	1.17 (1.11-1.23)
Multiple	10 312	5259 (51.0)	1.42	1.24 (1.22-1.27)	1.18 (1.15-1.22)	1.14 (1.10-1.17)

Abbreviation: HR, hazard ratio.

^a^ Model 1 adjusts for infant sex and year of birth and maternal age, parity, neighborhood income quintile, rurality, and immigrant status.

^b^ Model 2 additionally adjusts for maternal mental illness, substance use disorders, and stable and unstable chronic conditions.

Among infants with at least 1 ED visit, infants of women with an intellectual or developmental disability were less likely than infants of women without disabilities to have an outpatient visit with their regular primary care clinician within 7 days of their ED visit (RR, 0.85 [95% CI, 0.77-0.94]); however, the RR was nonsignificant after adjustment (adjusted RR, 0.96 [95% CI, 0.87-1.06]) (eTable 4 in Supplement 1). After adjustment, all disability groups except infants of women with multiple disabilities were more likely than women without disabilities to have a repeat ED visit (eg, adjusted RR, 1.16 [95% CI, 1.01-1.34] for intellectual or developmental disability) (eTable 5 in Supplement 1). Post hoc analyses revealed that, among infants with repeat ED visits, those of women with a disability were approximately as likely to have a pediatrician or family physician in a rostered primary care model, or to be part of a community health center (87.7% to 90.8% across groups), as infants of women without a disability (89.4%).

### Additional Analyses

In additional analyses, rates of ED visits in the first year of life, modeled as recurrent events, were higher among infants of women with disabilities (eTable 6 and the eFigure in Supplement 1). Findings were similar in analyses by disability subtype (eTable 7 in Supplement 1). Findings were also similar in analyses restricted to low-risk infants (eTable 8 in Supplement 1) and the pre–COVID-19 period (eTable 9 in Supplement 1).

## Discussion

In this population-based cohort study in a universal health care system, ED visits in infancy were common. Infants of women with a disability were more likely to have ED visits, particularly low-acuity visits, compared with infants of women without disabilities, although the characteristics of the ED visits, including timing, diagnosis, and discharge disposition, were similar across groups. Rates of follow-up with the primary care physician within 7 days were also similar. However, infants of women with a disability were more likely than infants of women without disabilities to have repeat ED visits.

Few studies have examined health care use among infants of women with a disability, with existing studies^16,17,18^ focusing on well-infant care and hospital admissions. A population-based study in Ontario previously found that infants of women with an intellectual or developmental disability were less likely than infants of women without a disability to receive routine well-infant visits and immunizations^16^; findings for other disability groups were nonsignificant. Using data from the UK Millennium Cohort Study, another study^17^ found no differences in immunizations but found elevated risk of hospital admission by 9 months comparing infants of mothers with or without an intellectual or developmental disability. Studies using prospective data from a US community clinic^18^ and Washington State health administrative data^19^ found that infants of women with an intellectual or developmental disability were more likely than infants of women without disabilities to be hospitalized by 2 years of age. These studies suggest possible outpatient health care access barriers and elevated health care needs among infants of women with an intellectual or developmental disability. Our study contributes to this literature by providing similar evidence from infant ED visits specifically.

Infant ED visits are strongly influenced by social determinants of health, with increases in ED visits in families experiencing poverty, racism, and other adversity.^3,4,7,8^ Women with a disability in the present cohort, and especially those with an intellectual or developmental disability, were more likely to experience poverty, and disparities in infant ED use were somewhat reduced after controlling for this and other factors. Neonatal complications such as preterm birth are also a known risk factor for infant ED use.^39^ Yet while infants of women with a disability are more likely to be born preterm,^40^ disparities in ED use remained in a cohort of low-risk infants in the present study, suggesting that other variables may contribute. For example, early infancy is a high-risk period for all families, with concerns such as feeding issues, jaundice, and infection that may require urgent attention.^1,3^ Our data suggest that infants of women with a disability have health concerns similar to those of women without a disability but are more likely to access the ED for these concerns. Contributing factors may include lack of maternal access to education about when concerns are urgent and where care should be accessed.^41^ Women with disabilities experience barriers to outpatient care, including physically inaccessible care environments, communication issues with clinicians, and negative clinician attitudes^42^; it is possible that these same barriers could affect access to primary care for their infants. A unique barrier to primary care for new parents, and especially those with intellectual or developmental disabilities, may also be fear of ableist clinicians’ beliefs about parenting and child welfare involvement.^43^ While there were few differences in attachment to primary care physicians in our cohort, such barriers might significantly impact the quality of outpatient care received. Ultimately, these barriers could result in mothers seeking ED care for their infants for nonurgent reasons or lead to health complications that necessitate urgent care.

Women with disabilities experience important structural and social disparities across the life course, so appropriate family supports should be put in place well before delivery. Health systems need to ensure equitable access to parental education about pediatric illnesses, how to manage low-acuity health concerns using a watch-and-wait approach, and how to recognize when urgent care is needed.^41^ Pediatricians, family physicians, and nurses may benefit from training to ensure pediatric outpatient care is accessible to and supportive of mothers with a disability so that families are supported and infant health concerns are identified and managed before they become urgent. Infants of women with a disability may also benefit from an extended newborn hospital stay or enhanced primary care or home visits in the first month^44^ to provide their families with social, educational, and breastfeeding supports. Finally, given that approximately half of infants of women with a disability had an ED visit and many returned to the ED, ED staff may require training on accessibility and support-related needs of mothers with a disability in acute care settings, with development of care plans to facilitate post-ED discharge communication with the regular health care clinician.^45^ Studies should also examine the experiences of families with repeat ED visits, including those with a very high number of ED visits. Such work should be done in consultation with people with a disability to ensure care meets their needs.

### Limitations

This study had limitations. Limitations included our reliance on diagnostic codes to measure maternal disability status. The disability algorithms, although frequently used,^24,26^ have not been tested for sensitivity, specificity, or predictive values. We likely misclassified disability status for women without a diagnosis or who had not sought health care for their disability.^46^ This would bias our results toward the null. We also had no data on disability severity. Furthermore, while we had data on ED visit acuity and discharge diagnosis, we could not definitively ascertain whether ED visits were preventable in outpatient settings nor other circumstances surrounding the decision to seek ED care, such as quality of the relationship with the primary care physician, transportation issues, or distance to care. Additionally, we had no data on individual-level income, maternal educational level, experiences of racism, or availability of social services and familial supports, all of which might impact maternal health care decisions and infant ED use. We also had no information on the quality of infant outpatient or ED care, which may have informed interpretation of our findings.

## Conclusions

This cohort study found that infants of women with a disability were more likely than infants of women without disabilities to have an ED visit and repeat ED visits, although the timing, diagnosis, discharge disposition, and rates of follow-up with the primary care physician within 7 days were similar. These findings suggest that more could be done for infants of women with a disability to prevent nonurgent ED visits, reduce risk factors for urgent ED visits, and support families when ED visits occur.

## Notes

Supplement 1. **eTable 1.** Description of ICES Datasets Used in the Analyses **eTable 2.** Standardized Differences for Table 1, Comparing Each Disability Group to No Disability **eTable 3.** Emergency Department Visit Characteristics, Including Timing During the Week, Discharge Diagnosis, and Discharge Disposition, Comparing Infants of Women With Various Types of Disabilities to Infants of Women Without a Disability **eTable 4.** Outpatient Visit With the Regular Primary Care Physician Within 7 Days of an Emergency Department (ED) Visit in the First Year of Life, Comparing Infants of Women With Various Types of Disabilities to Infants of Women Without a Disability **eTable 5.** Repeat Emergency Department (ED) Visit Within 7 Days of an Initial ED Visit in the First Year of Life, Comparing Infants of Women With Various Types of Disabilities to Infants of Women Without a Disability **eTable 6.** Rate of Emergency Department Use in the First Year of Life, Comparing Infants of Mothers With Various Types of Disabilities to Those Born to a Woman Without a Disability. **eFigure.** Cumulative Mean Number of Emergency Department (ED) Visits in the First Year of Life, Comparing Infants of Women With Various Types of Disability to Infants of Women Without a Disability **eTable 7.** Hazard of an Emergency Department (ED) Visit in the First Year of Life, Comparing Infants Born to Women With Various Types of Disabilities to Infants Born to Women Without a Disability, by Disability Subtype **eTable 8.** Hazard of an Emergency Department (ED) Visit in the First Year of Life, Comparing Infants Born to Women With Various Types of Disabilities to Infants Born to Women Without a Disability, Excluding Infants Born Preterm, Small for Gestational Age, With a Newborn Stay >72 hours, or With a Complex Chronic Condition **eTable 9.** Hazard of an Emergency Department Visit in the First Year of Life, Comparing Infants Born to Women With Various Types of Disabilities to Infants Born to Women Without a Disability, Restricted to Infants Born in 2008-2018

Supplement 2.
Data Sharing Statement
