# Supplementary material for: Maternal Disability and Emergency Department Use for Infants
Source: JAMA Netw Open. 2025 May 5;8(5):e258549. doi: 10.1001/jamanetworkopen.2025.8549 (PMC12053526; doi:10.1001/jamanetworkopen.2025.8549)
Supplement: Supplement 1. — eTable 1. Description of ICES Datasets Used in the Analyses eTable 2. Standardized Differences for Table 1, Comparing Each Disability Group to No Disability eTable 3. Emergency Department Visit Characteristics, Including Timing During the Week, Discharge Diagnosis, and Discharge Disposition, Comparing Infants of Women With Various Types of Disabilities to Infants of Women Without a Disability eTable 4. Outpatient Visit With the Regular Primary Care Physician Within 7 Days of an Emergency Department (ED) Visit in the First Year of Life, Comparing Infants of Women With Various Types of Disabilities to Infants of Women Without a Disability eTable 5. Repeat Emergency Department (ED) Visit Within 7 Days of an Initial ED Visit in the First Year of Life, Comparing Infants of Women With Various Types of Disabilities to Infants of Women Without a Disability eTable 6. Rate of Emergency Department Use in the First Year of Life, Comparing Infants of Mothers With Various Types of Disabilities to Those Born to a Woman Without a Disability. eFigure. Cumulative Mean Number of Emergency Department (ED) Visits in the First Year of Life, Comparing Infants of Women With Various Types of Disability to Infants of Women Without a Disability eTable 7. Hazard of an Emergency Department (ED) Visit in the First Year of Life, Comparing Infants Born to Women With Various Types of Disabilities to Infants Born to Women Without a Disability, by Disability Subtype eTable 8. Hazard of an Emergency Department (ED) Visit in the First Year of Life, Comparing Infants Born to Women With Various Types of Disabilities to Infants Born to Women Without a Disability, Excluding Infants Born Preterm, Small for Gestational Age, With a Newborn Stay >72 hours, or With a Complex Chronic Condition eTable 9. Hazard of an Emergency Department Visit in the First Year of Life, Comparing Infants Born to Women With Various Types of Disabilities to Infants Born to Women Without a Disability, Restricted to Infants Born in 2 [file jamanetwopen-e258549-s001.pdf]

Supplementary Online Content

Brown HK, Lunsky Y, Fung K, et al. Maternal disability and emergency department use for infants. *JAMA Netw Open*. 2025;8(5):e258549. doi:10.1001/jamanetworkopen.2025.8549

eTable 1. Description of ICES Datasets Used in the Analyses

eTable 2. Standardized Differences for Table 1, Comparing Each Disability Group to No Disability

eTable 3. Emergency Department Visit Characteristics, Including Timing During the Week, Discharge Diagnosis, and Discharge Disposition, Comparing Infants of Women With Various Types of Disabilities to Infants of Women Without a Disability

eTable 4. Outpatient Visit With the Regular Primary Care Physician Within 7 Days of an Emergency Department (ED) Visit in the First Year of Life, Comparing Infants of Women With Various Types of Disabilities to Infants of Women Without a Disability

eTable 5. Repeat Emergency Department (ED) Visit Within 7 Days of an Initial ED Visit in the First Year of Life, Comparing Infants of Women With Various Types of Disabilities to Infants of Women Without a Disability

eTable 6. Rate of Emergency Department Use in the First Year of Life, Comparing Infants of Mothers With Various Types of Disabilities to Those Born to a Woman Without a Disability

eFigure. Cumulative Mean Number of Emergency Department (ED) Visits in the First Year of Life, Comparing Infants of Women With Various Types of Disability to Infants of Women Without a Disability

eTable 7. Hazard of an Emergency Department (ED) Visit in the First Year of Life, Comparing Infants Born to Women With Various Types of Disabilities to Infants Born to Women Without a Disability, by Disability Subtype

eTable 8. Hazard of an Emergency Department (ED) Visit in the First Year of Life, Comparing Infants Born to Women With Various Types of Disabilities to Infants Born to Women Without a Disability, Excluding Infants Born Preterm, Small for Gestational Age, With a Newborn Stay >72 Hours, or With a Complex Chronic Condition

eTable 9. Hazard of an Emergency Department Visit in the First Year of Life, Comparing Infants Born to Women With Various Types of Disabilities to Infants Born to Women Without a Disability, Restricted to Infants Born in 2008-2018

© 2025 Brown HK et al. *JAMA Network Open*.

This supplementary material has been provided by the authors to give readers additional information about their work.

© 2025 Brown HK et al. *JAMA Network Open*.

eTable 1. Description of ICES datasets used in the analyses

Data source	Variable	Coding structure	Inception
Canadian Institute for Health Information Discharge Abstract Database	Hospital admissions	Canadian Coding Standards for the International Classification of Diseases and Related Health Problems codes for diagnoses and Canadian Classification of Health Interventions codes for procedures	1988
Client Agency Program Enrolment Database	Primary care enrollment	N/A	1999
Census	Sociodemographic data	N/A	2006, 2011, 2016
Immigrants, Refugees, and Citizenship Canada Permanent Residents Database	Immigration status	N/A	1985
National Ambulatory Care Reporting System	Emergency department visits	Canadian Coding Standards for the International Classification of Diseases and Related Health Problems codes for diagnoses and Canadian Classification of Health Interventions codes for procedures	2000
Ontario Health Insurance Database	Outpatient physician visits	Physician billing codes	1991
Ontario Mental Health Reporting System	Psychiatric hospital admissions	Diagnostic and Statistical Manual of Mental Disorders	2005
Registered Persons Database	Sociodemographic data	N/A	1991
Same Day Surgery database	Day surgeries	Canadian Coding Standards for the International Classification of Diseases and Related Health Problems codes for diagnoses and Canadian Classification of Health Interventions codes for procedures	1991

© 2025 Brown HK et al. *JAMA Network Open*.

eTable 2. Standardized differences for Table 1, comparing each disability group to no disability

**Variable [a]**	**Physical disability only** (N=139,698)	**Sensory disability only** (N=48,112)	**Intellectual/ developmental disability only** (N=2,547)	**Multiple disabilities** (N=10,312)
**Sociodemographic characteristics**				
Maternal age, years				
15-24	0.02	0.10	0.63	0.15
25-34	0.06	0.08	0.35	0.12
35-49	0.05	0.01	0.24	0.02
Multiparous	0.02	0.04	0.04	0.01
Neighborhood income quintile (Q)				
Q1 (lowest)	0.02	0.01	0.34	0.07
Q2	0.00	0.01	0.07	0.01
Q3	0.01	0.01	0.09	0.00
Q4	0.01	0.01	0.23	0.05
Q5 (highest)	0.02	0.00	0.17	0.04
Missing	0.01	0.01	0.03	0.01
Rural region of residence	0.11	0.05	0.08	0.10
Maternal immigrant status				
Recent refugee	0.11	0.11	0.11	0.15
Non-recent refugee	0.02	0.01	0.14	0.04
Recent immigrant	0.42	0.36	0.47	0.51
Non-recent immigrant	0.09	0.02	0.20	0.13
Long-term resident	0.41	0.30	0.56	0.51
Infant sex				
Male	0.00	0.00	0.00	0.00
Female	0.00	0.00	0.00	0.00
**Maternal health characteristics**				
Mental illness <2 years before delivery	0.19	0.13	0.62	0.35
Substance use disorder <2 years before delivery	0.10	0.02	0.31	0.18
Stable chronic condition <2 years before delivery	0.08	0.06	0.05	0.20
Unstable chronic conditions <2 years before delivery	0.14	0.10	0.15	0.29
**Infant health characteristics**				
Preterm birth	0.07	0.05	0.10	0.14
Small for gestational age	0.03	0.00	0.10	0.01

© 2025 Brown HK et al. *JAMA Network Open*.

Variable [a]	**Physical disability only** (N=139,698)	**Sensory disability only** (N=48,112)	**Intellectual/ developmental disability only** (N=2,547)	**Multiple disabilities** (N=10,312)
Birth hospital stay >72 hours	0.10	0.05	0.23	0.20
Complex chronic condition	0.05	0.04	0.15	0.13
**Healthcare characteristics**				
Maternal prenatal care adequacy				
None/unknown	0.00	0.00	0.01	0.01
Inadequate/intermediate	0.02	0.01	0.09	0.05
Adequate	0.04	0.04	0.14	0.11
Intensive	0.06	0.05	0.06	0.16
Infant regular primary care provider				
No regular primary care provider	0.01	0.02	0.04	0.01
General practitioner: no model	0.04	0.00	0.03	0.00
Pediatrician	0.07	0.01	0.06	0.05
General practitioner: rostered	0.08	0.01	0.02	0.03
Community health centre	0.01	0.02	0.12	0.05

© 2025 Brown HK et al. *JAMA Network Open*.

eTable 3. Emergency department visit characteristics, including timing during the week, discharge diagnosis, and discharge disposition, comparing infants of women with various types of disabilities to infants of women without a disability

Data reported as n (%)

**Variable**	**Physical disability only** (N=65,496)	**Sensory disability only** (N=21,744)	**Intellectual/ developmental disability only** (N=1,410)	**Multiple disabilities** (N=5,259)	**No disability** (N=558,965)
Timing during the week					
Business hours	44,874 (68.5)	14,983 (68.9)	957 (67.9)	3,612 (68.7)	388,081 (69.4)
After hours/weekends	20,622 (31.5)	6,761 (31.1)	453 (32.1)	1,647 (31.3)	170,884 (30.6)
Discharge diagnosis					
A00-B99 Certain infectious and parasitic diseases	7,127 (10.9)	2,326 (10.7)	148 (10.5)	521 (9.9)	60,335 (10.8)
C00-D48 Neoplasms	84 (0.1)	29 (0.1)	<6	11 (0.2)	659 (0.1)
D49-D89 Diseases of the blood and blood-forming organs	48 (0.1)	28 (0.1)	0 (0.0)	<6	497 (0.1)
E00-E90 Endocrine, nutritional and metabolic diseases	215 (0.3)	63 (0.3)	<6	9 (0.2)	1,767 (0.3)
F00-F99 Mental and behavioural disorders	39 (0.1)	9 (0.0)	0 (0.0)	8 (0.2)	335 (0.1)
G00-G99 Diseases of the nervous system	158 (0.2)	50 (0.2)	<6	19 (0.4)	1,231 (0.2)
H00-H59 Diseases of the eye and adnexa	1,354 (2.1)	487 (2.2)	24 (1.7)	126 (2.4)	10,822 (1.9)
H60-H95 Diseases of the ear and mastoid process	2,158 (3.3)	674 (3.1)	35 (2.5)	171 (3.3)	18,184 (3.3)
I00-I99 Diseases of the circulatory system	105 (0.2)	33 (0.2)	<6	10 (0.2)	820 (0.1)
J00-J99 Diseases of the respiratory system	16,129 (24.6)	5,136 (23.6)	320 (22.7)	1,232 (23.4)	132,703 (23.7)
K00-K93 Diseases of the digestive system	3,365 (5.1)	1,075 (4.9)	76 (5.4)	274 (5.2)	26,891 (4.8)
L00-L99 Diseases of the skin and subcutaneous tissue	2,110 (3.2)	723 (3.3)	56 (4.0)	191 (3.6)	18,276 (3.3)
M00-M99 Diseases of the musculoskeletal system and connective tissue	129 (0.2)	48 (0.2)	<6	<6	1,135 (0.2)
N00-N99 Diseases of the genitourinary system	1,063 (1.6)	387 (1.8)	20 (1.4)	89 (1.7)	10,227 (1.8)
P00-P96 Certain conditions originating in the perinatal Period	6,368 (9.7)	2,235 (10.3)	142 (10.1)	553 (10.5)	58,501 (10.5)
Q00-Q99 Congenital malformations, deformations and chromosomal abnormalities	526 (0.8)	170 (0.8)	13 (0.9)	43 (0.8)	3,902 (0.7)
R00-R99 Symptoms, signs and abnormal clinical and laboratory findings, not elsewhere classified	13,349 (20.4)	4,421 (20.3)	292 (20.7)	1,083 (20.6)	117,095 (20.9)
S00-T98 Injury, poisoning and certain other consequences of external causes	6,649 (10.2)	2,335 (10.7)	138 (9.8)	520 (9.9)	59,197 (10.6)
Other	4,520 (6.9)	1,515 (7.0)	132 (9.4)	388 (7.4)	36,388 (6.5)

© 2025 Brown HK et al. *JAMA Network Open*.

**Variable**	**Physical disability only** (N=65,496)	**Sensory disability only** (N=21,744)	**Intellectual/ developmental disability only** (N=1,410)	**Multiple disabilities** (N=5,259)	**No disability** (N=558,965)
Discharge disposition					
Admitted to hospital	6,479 (9.9)	2,205 (10.1)	162 (11.5)	515 (9.8)	53,703 (9.6)
Death	54 (0.1)	15 (0.1)	<6	<6	355 (0.1)
Discharged home	56,696 (86.6)	18,790 (86.4)	1,189 (84.3)	4,575 (87.0)	484,278 (86.6)
Left without being seen	2,164 (3.3)	705 (3.2)	51 (3.6)	160 (3.0)	19,574 (3.5)
Left without medical advice	103 (0.2)	29 (0.1)	<6	<6	1,055 (0.2)

© 2025 Brown HK et al. *JAMA Network Open*.

eTable 4. Outpatient visit with the regular primary care physician within 7 days of an emergency department (ED) visit in the first year of life, comparing infants of women with various types of disabilities to infants of women without a disability

Outpatient visit < 7 days after initial ED visit[a]	Number (%) with outcome	Unadjusted RR (95% CI)	Adjusted RR (95% CI)[b]
No disability (N=558,312)	142,422 (25.5)	1.00 (Referent)	1.00 (Referent)
Physical disability only (N=65,405)	16,195 (24.8)	0.97 (0.96-0.98)	1.01 (0.99-1.02)
Sensory disability only (N=21,720)	5,602 (25.8)	1.01 (0.99-1.04)	1.05 (1.02-1.07)
Intellectual/development disability only (N=1,405)	306 (21.8)	0.85 (0.77-0.94)	0.96 (0.87-1.06)
Multiple disabilities (N=5,255)	1,328 (25.3)	0.99 (0.94-1.04)	1.06 (1.01-1.11)

[a] Includes only infants who had an ED visit in the first year of life.

[b] Adjusts for infant sex, year of birth, and maternal age, parity, neighborhood income quintile, rurality, and immigrant status.

© 2025 Brown HK et al. *JAMA Network Open*.

eTable 5. Repeat emergency department (ED) visit within 7 days of an initial ED visit in the first year of life, comparing infants of women with various types of disabilities to infants of women without a disability

Repeat ED visit < 7 days after initial ED visit[a]	Number (%) with outcome	Unadjusted RR (95% CI)	Adjusted RR (95% CI)[b]
No disability (N=558,312)	55,407 (9.9)	1.00 (Referent)	1.00 (Referent)
Physical disability only (N=65,405)	7,095 (10.9)	1.09 (1.07-1.12)	1.07 (1.05-1.10)
Sensory disability only (N=21,720)	2,281 (10.5)	1.06 (1.02-1.10)	1.05 (1.01-1.09)
Intellectual/development disability only (N=1,405)	169 (12.0)	1.21 (1.05-1.40)	1.16 (1.01-1.34)
Multiple disabilities (N=5,255)	551 (10.5)	1.06 (0.97-1.15)	1.04 (0.95-1.12)

[a] Includes only infants who had an ED visit in the first year of life.

[b] Adjusts for infant sex, year of birth, and maternal age, parity, neighborhood income quintile, rurality, and immigrant status.

© 2025 Brown HK et al. *JAMA Network Open*.

eTable 6. Rate of emergency department use in the first year of life, comparing infants of mothers with various types of disabilities to those born to a woman without a disability

Disability type	Mean cumulative frequency	Rate per 1,000 person-days	Unadjusted RR (95% CI)	Adjusted RR (95% CI)[a]
No disability (N=1,396,263)	0.38	2.08	1.00 (Referent)	1.00 (Referent)
Physical only (N=139,698)	0.52	2.75	1.38 (1.37-1.40)	1.34 (1.32-1.35)
Sensory only (N=48,112)	0.49	2.57	1.27 (1.25-1.30)	1.23 (1.21-1.25)
Intellectual/development only (N=2,547)	0.81	3.95	2.13 (1.97-2.30)	1.72 (1.59-1.85)
Multiple (N=10,312)	0.68	3.35	1.76 (1.69-1.83)	1.65 (1.59-1.72)

Abbreviations: CI = confidence interval; RR = rate ratio.

[a] Model adjusts for infant sex, year of birth, and maternal age, parity, neighborhood income quintile, rurality, and immigrant status.

© 2025 Brown HK et al. *JAMA Network Open*.

eFigure. Cumulative mean number of emergency department (ED) visits in the first year of life, comparing infants of women with various types of disability to infants of women without a disability
-- = value suppressed to prevent reidentification of individuals.

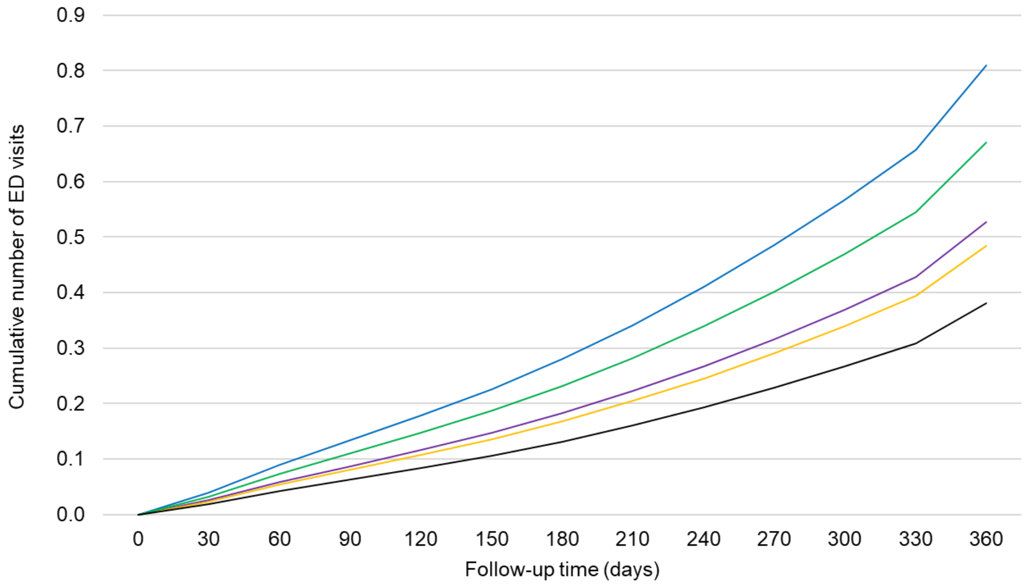

No. at risk

Physical disability	139,698	139,523	139,416	139,263	139,057	138,465	126,238
Sensory disability	48,112	48,068	48,025	47,982	47,923	47,739	43,918
IDD	2,547	2,540	2,530	--	2,509	2,487	2,185
Multiple disabilities	10,312	10,304	10,292	10,283	10,270	10,208	8,976
No disability	1,396,263	1,394,764	1,393,223	1,391,628	1,389,445	1,383,964	1,291,767

© 2025 Brown HK et al. *JAMA Network Open*.

eTable 7. Hazard of an emergency department (ED) visit in the first year of life, comparing infants born to women with various types of disabilities to infants born to women without a disability, by disability subtype

Disability type	Number (%) with outcome	Incidence per 1,000 person-days	Unadjusted HR (95% CI)	Adjusted HR (95% CI)[a]
No disability N=1,396,263)	558,965 (40.0)	1.11	1.00 (Referent)	1.00 (Referent)
Physical				
Congenital anomaly exclusively (N=11,606)	5,595 (48.2)	1.34	1.18 (1.15-1.20)	1.09 (1.06-1.12)
Musculoskeletal disorder exclusively (N=59,180)	26,303 (44.5)	1.23	1.11 (1.10-1.12)	1.12 (1.11-1.14)
Neurological injury exclusively (N=34,973)	16,512 (47.2)	1.31	1.17 (1.16-1.19)	1.14 (1.12-1.16)
Permanent injury exclusively (N=22,804)	11,345 (49.8)	1.38	1.23 (1.21-1.25)	1.15 (1.13-1.18)
Multiple physical disabilities (N=11,135)	5,741 (51.6)	1.43	1.27 (1.25-1.30)	1.24 (1.20-1.27)
Sensory				
Hearing impairment exclusively (N=33,384)	15,576 (46.7)	1.29	1.15 (1.13-1.16)	1.10 (1.08-1.11)
Vision impairment exclusively (N=14,191)	5,920 (41.7)	1.16	1.02 (1.00-1.04)	1.06 (1.03-1.09)
Multiple sensory disabilities (N=537)	248 (46.2)	1.28	1.14 (1.03-1.26)	1.14 (1.01-1.29)
Intellectual/developmental				
Autism exclusively (N=537)	296 (55.1)	1.54	1.36 (1.26-1.47)	1.18 (1.05-1.32)
Intellectual disability exclusively (N=1,976)	1,095 (55.4)	1.55	1.38 (1.32-1.44)	1.25 (1.18-1.33)
Multiple IDD (N=34)	19 (55.9)	1.61	1.43 (1.06-1.92)	1.29 (0.92-1.80)
Multiple disabilities				
Physical and Sensory (N=9,134)	4,552 (49.8)	1.38	1.21 (1.19-1.24)	1.18 (1.14-1.21)
Physical and IDD (N=748)	440 (58.8)	1.65	1.45 (1.36-1.55)	1.19 (1.08-1.31)
Sensory and IDD (N=279)	158 (56.6)	1.59	1.40 (1.25-1.57)	1.15 (0.98-1.35)
All three types of disabilities (N=151)	109 (72.2)	2.02	1.77 (1.58-1.98)	1.63 (1.34-2.00)

IDD = intellectual/developmental disability

[a] Adjusts for infant sex, year of birth, and maternal age, parity, neighborhood income quintile, rurality, and immigrant status.

© 2025 Brown HK et al. *JAMA Network Open*.

eTable 8. Hazard of an emergency department (ED) visit in the first year of life, comparing infants born to women with various types of disabilities to infants born to women without a disability, excluding infants born preterm, small for gestational age, with a newborn stay >72 hours, or with a complex chronic condition

Disability type	Number (%) with outcome	Incidence per 1,000 person-days	Unadjusted HR (95% CI)	Adjusted HR (95% CI)[a]
No disability (N=985,521)	382,516 (38.81)	1.07	1.00 (Referent)	1.00 (Referent)
Physical disability only (N=94,763)	42,841 (45.21)	1.25	1.16 (1.15-1.17)	1.13 (1.12-1.14)
Sensory disability only (N=33,048)	14,527 (43.96)	1.21	1.11 (1.10-1.13)	1.09 (1.07-1.11)
Intellectual/development disability only (N=1,531)	836 (54.60)	1.51	1.39 (1.32-1.46)	1.27 (1.19-1.36)
Multiple disabilities (N=6,466)	3,138 (48.53)	1.34	1.21 (1.18-1.25)	1.18 (1.13-1.22)

[a] Adjusts for infant sex, year of birth, and maternal age, parity, neighborhood income quintile, rurality, and immigrant status.

© 2025 Brown HK et al. *JAMA Network Open*.

eTable 9. Hazard of an emergency department visit in the first year of life, comparing infants born to women with various types of disabilities to infants born to women without a disability, restricted to infants born in 2008-2018

Disability type	Number (%) with outcome	Incidence per 1,000 person-days	Unadjusted HR (95% CI)	Adjusted HR (95% CI)[a]
No disability (N=1,193,520)	491,428 (41.2)	1.14	1.00 (Referent)	1.00 (Referent)
Physical disability only (N=118,052)	57,047 (48.3)	1.34	1.16 (1.15-1.17)	1.13 (1.12-1.14)
Sensory disability only (N=39,296)	18,463 (47.0)	1.30	1.11 (1.10-1.12)	1.08 (1.07-1.10)
Intellectual/development disability only (N=2,020)	1,172 (58.0)	1.62	1.39 (1.33-1.45)	1.23 (1.16-1.30)
Multiple disabilities (N=8,219)	4,385 (53.4)	1.48	1.25 (1.22-1.28)	1.18 (1.14-1.21)

[a] Adjusts for infant sex, year of birth, and maternal age, parity, neighborhood income quintile, rurality, and immigrant status.

© 2025 Brown HK et al. *JAMA Network Open*.
