# Supplementary material for: Maternal Disability and Emergency Department Use for Infants
Source: JAMA Netw Open. 2025 May 5;8(5):e258549. doi: 10.1001/jamanetworkopen.2025.8549 (PMC12053526; doi:10.1001/jamanetworkopen.2025.8549)
Supplement: Supplement 2. — Data Sharing Statement [file jamanetwopen-e258549-s002.pdf]

Data Sharing Statement

Brown. Maternal Disability and Emergency Department Use for Infants. *JAMA Netw Open*. Published May 05, 2025. doi:10.1001/jamanetworkopen.2025.8549

Data
Data available: No

Additional Information
Explanation for why data not available: Data used for this study were housed at ICES, an independent not-for-profit corporation. While data sharing agreements prohibit ICES from making the data set publicly available, access can be granted to those who to meet pre-specified criteria for confidential access, available at www.ices.on.ca/. Requests to access ICES data for research purposes may be submitted to ICES' Data and Analytic Services. Visit http://www.ices.on.ca/DAS for more information, including contact details.
